# Cross‐Cultural Adaptation of the Pool Activity Level (PAL) Checklist: From UK English to U.S. English for Enhanced Dementia Care

**DOI:** 10.1002/alz.089474

**Published:** 2025-01-09

**Authors:** Shadi Gholizadeh, Liz Sudberry, Elizabeth M. Popolizio, Erin L Merz, Damaris Daniels, Jackie Pool

**Affiliations:** ^1^ TheKey Research Group, San Diego, CA USA; ^2^ THEKEY Research Group, Cleveland, OH USA; ^3^ Cal State University Dominguez Hills, Dominguez Hills, CA USA; ^4^ Quality Compliance Systems, High Wycombe, NA United Kingdom; ^5^ Dementia Pal Ltd, Southampton United Kingdom

## Abstract

**Background:**

An important aspect of quality of life is engagement in meaningful and purposeful activities. For people living with dementia (PLwD), opportunities to engage in purposeful activities can be vastly diminished. Successful engagement of individuals with dementia typically hinges upon selecting engagement targets and activities that are appropriate to the individual’s level of cognitive functioning. The Pool Activity Levels (PAL) Checklist, a strength‐based, person‐centered 9‐item checklist tool for nonclinical caregivers to assess cognitive, physical, and sensory abilities in PLwD in order to guide activity planning and engagement strategies, was originally developed in British English. The increasing use of the measure in the United States necessitates a translation into American English to ensure cultural and linguistic appropriateness. Back translation (BT) is a commonly‐used methodology of questionnaire translation. However, several problems with BT have been highlighted, including its reliance on perfect equivalence and prioritization of literal translation over conceptual and cultural equivalence. Alternatives to BT include engaging professionals with expertise in translation and the subject matter to translate the assessment and to then submit the translation to a review by an expert panel and field test or using an iterative, team based approach that involves translation, review, evaluation, pretesting, and documentation.

**Method:**

Adopting a multidisciplinary approach, the PAL Checklist was translated from British to U.S. English. This process involved a team of professionals, including collaboration with the original author of the PAL, from various U.S. regions with expertise in care planning and assessment. The translation methodology aligned with the ISPOR Quality of Life Special Interest Group guidelines, incorporating steps like forward translation, reconciliation, back translation, cognitive debriefing, and finalization.

**Result:**

The translation retained the original checklist’s conceptual integrity while adapting linguistic differences, such as “flannel” to “washcloth/sponge” and “cupboards” to “drawers/closet.”

**Conclusion:**

This preliminary translation of the PAL into U.S. English lays the groundwork for future research to assess its psychometric properties in American settings. Future research will focus on extensive validation to meet psychometric criteria (e.g., internal consistency reliability, structural validity), ensuring its efficacy and reliability in the United States.

